# The Impact of Stigma on Autonomous Motivation Among Psychiatric Hospital Staff in China: The Mediating Role of Turnover Intention

**DOI:** 10.1155/jonm/9940890

**Published:** 2026-03-18

**Authors:** Yaning Yin, Luping Hei, Miaomiao Zhao, Siwei Sun, Beibei Yuan

**Affiliations:** ^1^ Planning Department, Shandong Victory Information Technology Co., Ltd., Jinan, Shandong, China; ^2^ University and College Affairs Department, Zhengzhou Bureau of Science and Technology, Zhengzhou, Henan, China; ^3^ Shanghai Mental Health Center, Shanghai Jiao Tong University, Shanghai, China, sjtu.edu.cn; ^4^ Center for Mental Health Management, China Hospital Development Institute (Shanghai Jiao Tong University), Shanghai, China; ^5^ Peking University Sixth Hospital, Peking University, Beijing, China, pku.edu.cn; ^6^ China Center for Health Development Studies, Peking University, Beijing, China, pku.edu.cn

**Keywords:** autonomous motivation, self-determination theory, stigma, turnover intention

## Abstract

**Objectives:**

Stigma is a persistent psychosocial stressor in psychiatric care settings, yet its impact on the motivation of nursing staff and other healthcare professionals remains underexplored. This study examined how four dimensions of stigma—perceived stigma, self‐stigma, social concealment, and positive response—relate to autonomous motivation and whether turnover intention mediates these relationships.

**Methods:**

A cross‐sectional survey was conducted with 2813 employees from 36 psychiatric hospitals across six provinces in China. Validated scales measured stigma dimensions, turnover intention, and autonomous motivation. Structural equation modeling with bootstrapped confidence intervals tested direct and indirect pathways.

**Results:**

Perceived stigma directly predicted lower autonomous motivation, whereas self‐stigma and social concealment reduced motivation only indirectly via turnover intention. Notably, since the positive response dimension was reverse‐scored, higher stigma scores (representing lower positive response) were associated with reduced autonomous motivation through both direct and indirect pathways. Perceived stigma showed no significant indirect effect through turnover intention.

**Conclusions:**

Different stigma dimensions influence motivation through distinct psychological mechanisms. Strategies that reduce internalized stigma, foster value‐affirming behaviors, and address turnover‐related strain may help sustain autonomous motivation in high‐pressure psychiatric settings. These findings offer actionable insights for nursing managers seeking to strengthen workforce engagement and retention.

## 1. Introduction

Mental disorders constitute a major public health challenge in China. In 2019, more than 160 million adults were affected, with a 12‐month prevalence of 9.3% and a lifetime prevalence of 16.6% [1, 2], according to the national epidemiological survey on mental health in China. The World Health Organization has identified strengthening the mental health workforce as a global priority [3]. In China, psychiatric hospitals continue to experience staffing shortages, making staff stability and motivation critical to sustaining high‐quality care [4, 5]. Against this backdrop, nurses represent a substantial proportion of the psychiatric workforce and frequently undertake both direct clinical care and coordination tasks, underscoring the importance of understanding motivational processes relevant to nursing and human resource management in psychiatric settings.

Self‐determination theory (SDT) conceptualizes motivation through the satisfaction of three basic psychological needs—autonomy, competence, and relatedness [6–8]. Autonomous motivation, encompassing identified and intrinsic regulation, is particularly important in high‐pressure care environments, as it is associated with proactive engagement and better quality of care [9–13]. However, psychiatric hospital staff may encounter occupational stigma, which may undermine these needs and, consequently, autonomous motivation.

Occupational stigma refers to negative societal beliefs, attitudes, and behaviors directed at a profession [14, 15]. For psychiatric hospital staff, stigma has been conceptualized as a multidimensional construct, including perceived stigma (awareness of public negativity), self‐stigma (internalized negative beliefs), social concealment (hiding one’s occupation), and positive response [16, 17]. In this context, “positive response” reflects whether staff take affirmative actions in daily life to address public misunderstandings about their work and to improve the social perception of their profession. Existing evidence has linked stigma to adverse work outcomes such as burnout, lower job satisfaction, and stronger turnover intention [18–21]. Nevertheless, less is known about how distinct stigma dimensions relate to positive motivational constructs such as autonomous motivation in psychiatric hospital settings and whether these associations operate through proximal withdrawal‐related cognitions.

Turnover intention, defined as the conscious decision to leave an organization, represents a central withdrawal‐related belief and a key cognitive precursor to disengagement [22, 23]. Drawing on SDT and the affective–cognitive–behavioral (ABC) model, stigma may function as an activating event that shapes such beliefs, which in turn influence subsequent emotional and motivational processes [24–26]. Specifically, elevated turnover intention may undermine autonomous motivation by interrupting the internalization of work‐related values, weakening psychological attachment to professional roles, and frustrating basic psychological needs for autonomy, competence, and relatedness. Prior studies have linked stigma to withdrawal‐related cognitions and disengagement tendencies among psychiatric staff, suggesting that turnover intention may serve as a critical cognitive pathway through which stigma exerts its motivational consequences [27]. However, the interplay between stigma, turnover intention, and autonomous motivation remains underexplored in psychiatric contexts.

Therefore, this study aims to investigate stigma among psychiatric hospital staff, with a particular focus on the nursing workforce. Given that psychiatric care relies on multidisciplinary collaboration, examining nurses within the broader hospital context rather than in isolation provides a more ecologically valid understanding of their occupational environment. To address existing gaps, this study specifically examines how four dimensions of occupational stigma relate to autonomous motivation, with turnover intention proposed as a mediator. The hypotheses are as follows:

i. Perceived stigma affects autonomous motivation both directly and indirectly through turnover intention;
ii. Self‐stigma affects autonomous motivation directly and indirectly through turnover intention;
iii. Social concealment affects autonomous motivation directly and indirectly through turnover intention; and
iv. Positive responses affect autonomous motivation directly and indirectly through turnover intention.

## 2. Methods

### 2.1. Participants and Procedures

A cross‐sectional survey was conducted between March and October 2023. To ensure the sample’s representativeness across China’s vast geographic and socioeconomic landscape, we employed a multistage, stratified, cluster‐based sampling framework. The sampling process was organized into three stages.

 Stage 1 (geographic stratification). We purposively selected six provincial‐level administrative regions to capture heterogeneity in economic development and geographic location. These included Beijing Municipality (representing the developed capital economic circle); Shandong and Fujian Provinces (Eastern coastal region); Henan Province (Central China); and Inner Mongolia Autonomous Region and Guizhou Province (Western China). This selection ensures a balanced coverage of the nation’s eastern, central, and western development belts.
 Stage 2 (provincial clusters). In each selected region, the leading provincial‐level tertiary psychiatric specialty hospital was included. These six hospitals represent the highest level of psychiatric care and academic authority within their respective provinces.
 Stage 3 (municipal stratification and selection). To ensure socioeconomic diversity, municipal administrative units within each province were stratified based on local gross domestic product (GDP). Five cities were selected per province to cover the upper, middle, and lower tertiles of economic development. Within each selected city, one public municipal psychiatric hospital was recruited based on institutional feasibility.

In total, 36 psychiatric hospitals (6 provincial‐level and 30 municipal‐level) participated.

### 2.2. Ethical Approval and Informed Consent

The study protocol was approved by the Biomedical Ethics Committee of Peking University (IRB00001052‐22175). Data collection was officially coordinated by the Peking University Sixth Hospital, ensuring high institutional engagement through official correspondence and coordinator training.

The survey was administered via an online survey platform. To ensure standardized data collection, nursing departments and hospital administrators facilitated the distribution of the survey link during shift handovers or staff meetings. Prior to accessing the items, participants were presented with an electronic informed consent form outlining the study’s voluntary nature and their right to withdraw at any time. Participation only commenced once the individual clicked on the “I Agree” button.

### 2.3. Recruitment and Eligibility

Within each selected hospital, a census‐based invitation strategy was employed rather than random sampling of individuals. This approach was chosen to maximize the sample size and capture the full organizational climate. A designated liaison officer in each hospital distributed the survey link to all multidisciplinary staff via internal administrative systems. The inclusion criteria were as follows: (i) current employment in the target hospital; (ii) age ≥ 18 years; and (iii) ≥ 1 year of professional experience. Participation was entirely voluntary and anonymous.

### 2.4. Sample Size Design

The structural equation model included 25 observed indicators (15 for stigma, 4 for turnover intention, and 6 for autonomous motivation). Determination of the required sample size was guided by rigorous psychometric standards to ensure statistical power and solution stability. Specifically, Stevens [28] advocates that the sample size should be at least 15 times the number of observed variables, while Field [29] suggests a minimum absolute sample size of 300 for stable convergence. Based on the strict 15:1 ratio, the theoretical minimum sample size required was 375 (25 × 15). Additionally, to accommodate the cluster sampling design across 36 hospitals, we aimed for a target of at least 50 responses per institution to ensure sufficient representativeness within each cluster. The final valid sample of 2813 (averaging approximately 78 per hospital) substantially exceeds these theoretical requirements, ensuring robust statistical power for complex model evaluation.

### 2.5. Participant Characteristics

The study population comprised the multidisciplinary workforce within the participating institutions to capture the holistic organizational climate in which care is delivered. Registered nurses constituted the majority of the final valid sample (*N* = 2813), accounting for 52.04% of participants. The remaining sample included 28.83% licensed (assistant) physicians, 3.80% traditional Chinese medicine physicians, and 15.32% other professionals (e.g., administrative or allied health staff).

Regarding professional characteristics, 48.95% of participants reported a nursing background, while 30.61% had a psychiatry‐related background. In terms of employment status, 57.27% were employed in established posts, while 42.73% were nonestablished staff. Additionally, 13.54% held senior professional titles. This composition reflects the collaborative reality of mental health services, ensuring that the investigation of stigma is grounded in the authentic working environment of the nursing staff.

### 2.6. Data Quality Control and Screening

The survey initially received 3620 submissions. To ensure the reliability of the structural equation modeling (SEM) analysis, a rigorous data cleaning protocol was implemented. Responses were excluded (*n* = 807, 22.3%) based on the following predefined criteria.

#### 2.6.1. Demographic and Logical Inconsistencies (*n* = 84)

Specifically, 16 cases were removed for reporting biologically impossible ages (e.g., 130, 448, or 2525 years) or being under the professional eligibility age of 18. Additionally, 2 cases were excluded where the reported professional tenure exceeded the participant’s chronological age, and 66 cases involving extreme annual income outliers were removed as they were statistically inconsistent with the professional role.

#### 2.6.2. Attention Checks and Response Integrity (*n* = 723)

The majority of exclusions involved participants who failed either of the two embedded logic‐verification items (e.g., failing to select a specific predefined option such as “*Very Dissatisfied*” when instructed) or exhibited invariant response patterns (e.g., “straight‐lining” across Likert‐scale items).

Following this screening, 2813 valid questionnaires were retained for analysis, yielding an effective response rate of 77.7%.

### 2.7. Measures

#### 2.7.1. Stigma

In China, we assessed the stigma experienced by psychiatric hospital staff using a 15‐item occupational stigma scale [17]. The scale demonstrates good reliability and validity, comprising four dimensions: perceived stigma (five items), self‐stigma (three items), social masking (three items), and positive reactions (four items). Participants responded on a 7‐point scale, with the four items in the positive response dimension being reverse‐scored, with higher scores indicating higher stigma (i.e., lower levels of positive response). Total scores range from 15 to 105, with higher scores indicating greater levels of stigma. Overall Cronbach’s alpha coefficient for the scale was 0.904.

#### 2.7.2. Turnover Intention

The turnover intention scale consists of four items rated on a 7‐point Likert scale, where higher scores indicate a greater agreement with the statements. This scale primarily measures the respondent’s willingness to leave the organization, the industry, and their intention to seek new employment in the near future [30]. Cronbach’s alpha coefficient in this study was 0.945.

#### 2.7.3. Autonomous Motivation

The autonomous motivation scale, a subscale of the Chinese version of the Work Motivation Scale, consists of six questions, incorporating two dimensions of intrinsic regulation (Questions 1 to 3) and identified regulation (Questions 4 to 6), using an 11‐point Likert scale ranging from 0 to 10. Higher scores reflect a greater degree of compliance [31]. Cronbach’s alpha coefficient for autonomous motivation in this study was 0.961 following the reliability test.

### 2.8. Statistical Analysis

Data analysis was performed using SPSS 26.0 and AMOS 26.0. Descriptive statistics were computed to summarize participant characteristics, and data distribution was assessed using skewness and kurtosis.

#### 2.8.1. Measurement Model Evaluation

Confirmatory factor analysis (CFA) was conducted to assess the psychometric properties of the scales. Convergent validity was evaluated using composite reliability (CR) and average variance extracted (AVE) [32]. Discriminant validity was assessed using the Fornell–Larcker criterion [32] (comparing the square root of AVE with interconstruct correlations).

#### 2.8.2. Control for Common Method Bias (CMB)

To address potential CMB inherent in self‐report data, a CFA with an unmeasured common latent factor (CLF) approach was employed [33]. A CLF was added to the measurement model with paths specified to all observed indicators. Model fit indices and standardized regression weights were compared between the model with and without the CLF to assess the impact of method variance.

#### 2.8.3. Structural Model and Hypothesis Testing

SEM with maximum likelihood estimation was used to test the hypothesized relationships. Model fit was evaluated using established indices: chi‐square/df ratio (*χ* ^2^/df < 5), comparative fit index (CFI > 0.90), Tucker–Lewis index (TLI > 0.90), standardized root mean square residual (SRMR < 0.08), and root mean square error of approximation (RMSEA < 0.08) [34, 35].

Indirect effects were tested using bias‐corrected bootstrapping with 5000 resamples to generate 95% confidence intervals (CIs) [36]. Given the cross‐sectional design, path coefficients are interpreted as predictive associations rather than causal inferences. Statistical significance was defined as a two‐tailed *p* < 0.05.

## 3. Results

### 3.1. Participants’ Characteristics

A total of 2813 valid questionnaires were collected (see Table 1). The participants were predominantly female (71.17%) with a mean age of 35.84 years (SD = 8.40). Notably, registered nurses constituted the majority of the sample (52.04%), ensuring the study’s relevance to the nursing workforce context.

**TABLE 1:** Demographic characteristics of study participants (*n* = 2813).

Variables	Classification	*n*	%
Sex	Male	811	28.83
	Female	2002	71.17

Age (years) (Mean = 35.84, SD = 8.40)	< 30	687	24.42
	30–40	1445	51.37
	> 40	681	24.21

Marital status	Married	2061	73.27
	Unmarried	752	26.73

Education level	Master’s degree or above	310	11.02
	Bachelor’s degree	2007	71.35
	College degree or below	496	17.63

Professional background (discipline)	Nursing	1377	48.95
	Psychiatry/mental health	861	30.61
	Other	575	20.44

Professional qualification (license)	Registered nurse	1464	52.04
	Physician/assistant physician	811	28.83
	TCM physician	107	3.80
	Other	431	15.32

Professional title	Associate senior or above	381	13.54
	Intermediate	917	32.60
	Junior or below	1515	53.86

Administrative position	Yes	487	17.31
	No	2326	82.69

Employment status	Established	1611	57.27
	Contract‐based	1202	42.73

Work experience	< 10 years	1281	45.54
	10–20 years	1079	38.36
	> 20 years	453	16.10

Annual income (CNY)	< 80,000	968	34.41
	80,000–150,000	1422	50.55
	> 150,000	423	15.04

Hospital level	Tertiary	1900	67.54
	Secondary	913	32.46

Daily working hours	≤ 8 h	1980	70.39
	> 8 h	833	29.61

### 3.2. Descriptive Statistics and Measurement Model Assessment

Prior to model testing, data normality was assessed. Skewness values for all items were less than 3, and kurtosis values were less than 8, indicating that the data did not substantially deviate from a normal distribution. Table 2 presents the descriptive statistics, psychometric properties, and correlations of the study variables. The mean scores for the four dimensions of occupational stigma were as follows: perceived stigma (19.40 ± 8.47), self‐stigma (8.75 ± 4.57), social concealment (11.19 ± 4.75), and positive response (11.19 ± 4.50).

**TABLE 2:** Descriptive statistics, psychometric properties, and correlations (*N* = 2813).

Variables	Mean	SD	CR	AVE	1	2	3	4	5	6
1. Perceived stigma	19.40	8.47	0.97	0.85	**(0.92)**					
2. Self‐stigma	8.75	4.57	0.88	0.72	0.42^∗∗^	**(0.85)**				
3. Social concealment	11.19	4.75	0.91	0.78	0.23^∗∗^	0.49^∗∗^	**(0.89)**			
4. Positive response	11.19	4.50	0.82	0.55	0.35^∗∗^	0.60^∗∗^	0.35^∗∗^	**(0.74)**		
5. Turnover intention	10.50	5.93	0.94	0.80	0.27^∗∗^	0.55^∗∗^	0.36^∗∗^	0.59^∗∗^	**(0.90)**	
6. Autonomous motivation	45.22	11.85	0.95	0.77	−0.27^∗∗^	−0.43^∗∗^	−0.24^∗∗^	−0.57^∗∗^	−0.56^∗∗^	**(0.88)**

*Note:* Diagonal elements in bold parentheses () represent the square root of AVE; off‐diagonal elements represent the correlations between constructs.

Abbreviations: AVE = average variance extracted, CR = composite reliability, SD = standard deviation.

^∗∗^ *p* < 0.01 (two‐tailed).

The initial CFA yielded suboptimal fit indices (*χ* ^2^/df = 23.20, RMSEA = 0.089, and CFI = 0.924), suggesting the need for model refinement. Respecification was performed based on modification indices (MIs) and substantive theoretical considerations. First, residual covariances were freed for item pairs within the same construct that exhibited clear semantic redundancy to control for method effects.

Specifically, residual covariances were allowed between Item A5 (perceived stigma) and self‐stigma indicators to capture the “media internalization effect.” As Item A5 references negative media portrayals, a potent source of societal devaluation [37], it serves as a direct cognitive antecedent to the professional inferiority measured by self‐stigma. Correlating these errors thus controls for shared variance arising from this specific internalization mechanism. Finally, residual correlations within autonomous motivation items were included based on SDT [7].

**FIGURE 1:**
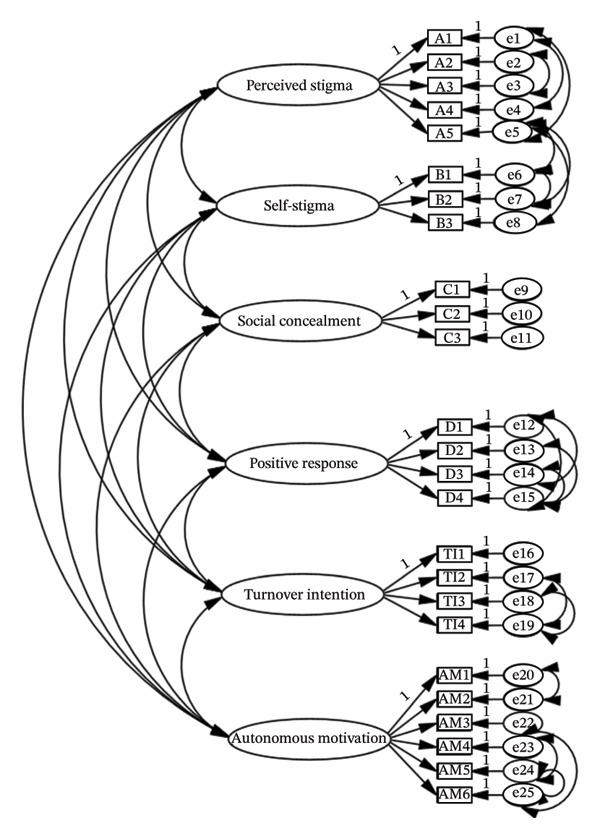
Modified confirmatory factor analysis model.

The revised measurement model demonstrated a substantially improved and acceptable fit (Figure 1): *χ* ^2^/df = 4.68, RMSEA = 0.036, CFI = 0.988, TLI = 0.985, and SRMR = 0.033. All standardized factor loadings were statistically significant (*p* < 0.001).

As summarized in Table 2, convergent validity was established as all CR values ranged from 0.82 to 0.97 (> 0.70), and AVE values exceeded 0.50. Discriminant validity was confirmed, as the square root of the AVE for each construct (bold diagonal elements) was greater than the interconstruct correlations. Correlation analysis revealed that turnover intention was positively correlated with all stigma dimensions (*r* = 0.27 to 0.59, *p* < 0.01), whereas autonomous motivation showed significant negative correlations with these dimensions (*r* = −0.24 to −0.57, *p* < 0.01).

### 3.3. CMB Test

Given the cross‐sectional nature of the data, potential CMB was assessed using the unmeasured latent method factor (ULMC) approach [33]. A CLF was added to the baseline measurement model, with paths connected to all observed indicators.

The CLF model yielded the following fit indices: *χ* ^2^/df = 3.05, CFI = 0.994, TLI = 0.992, RMSEA = 0.027 (90% CI [0.025, 0.029]), and SRMR = 0.019. While the inclusion of the CLF resulted in a statistically better fit compared to the baseline model (Δ CFI = 0.006; Δ RMSEA = −0.009; and Δ SRMR = −0.014), the changes were marginal and below the specific threshold of 0.05 for substantial model improvement [38, 39].

Although some variance was captured by the method factor, the structural relationships and factor loadings in the baseline model remained robust. This indicates that while method effects exist, they do not substantially distort the hypothesized relationships or the interpretation of the findings. Thus, CMB was not considered a pervasive threat in this study.

### 3.4. Structural Model and Hypothesis Testing

**FIGURE 2:**
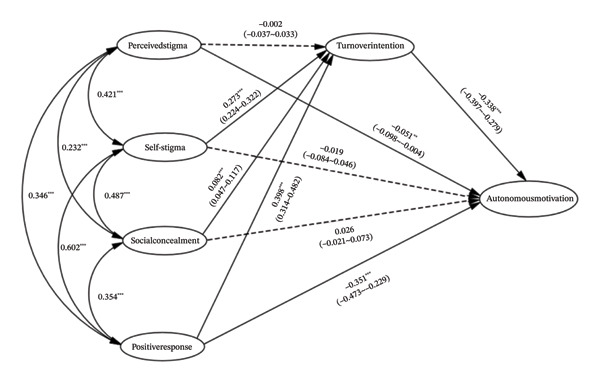
Structural equation modeling of perceived stigma, self‐stigma, social concealment, positive response, and autonomous motivation.

The structural model was assessed using the maximum likelihood estimation method. The model exhibited an excellent fit to the data: *χ* ^2^/df = 4.68, RMSEA = 0.036, CFI = 0.988, TLI = 0.985, and SRMR = 0.033. The SEM results indicating the relationships among perceived stigma, self‐stigma, social concealment, positive response, turnover intention, and autonomous motivation, along with the standardized path coefficients, are shown in Figure 2. The detailed coefficients and significance levels are further presented in Table 3.

**TABLE 3:** Standardized path coefficients and significance tests.

Paths	Estimate	S.E.	C.R.	*P*
Turnover intention <‐‐‐ perceived stigma	−0.002	0.018	−0.112	0.911
Turnover intention <‐‐‐ self‐stigma	0.273	0.025	11.171	^∗∗∗^
Turnover intention <‐‐‐ social concealment	0.082	0.018	4.415	^∗∗∗^
Turnover intention <‐‐‐ positive response	0.398	0.043	15.207	^∗∗∗^
Autonomous motivation <‐‐‐ turnover intention	−0.338	0.03	−15.284	^∗∗∗^
Autonomous motivation <‐‐‐ perceived stigma	−0.051	0.024	−2.938	0.003^∗∗^
Autonomous motivation <‐‐‐ self‐stigma	−0.019	0.033	−0.798	0.425
Autonomous motivation <‐‐‐ positive response	−0.351	0.062	−12.719	^∗∗∗^
Autonomous motivation <‐‐‐ social concealment	0.026	0.024	1.439	0.150

^∗∗^ *p* < 0.01.

^∗∗∗^ *p* < 0.001.

#### 3.4.1. Direct Effects

Regarding turnover intention, the analysis showed that self‐stigma (*β* = 0.27, *p* < 0.001) and social concealment (*β* = 0.08, *p* < 0.001) had significant positive effects. Notably, the positive response dimension, which was reverse‐scored (i.e., higher scores indicate higher stigma), showed a significant positive effect on turnover intention (*β* = 0.40, *p* < 0.001). This indicates that lower levels of positive coping strategies significantly predict higher turnover intention. In contrast, perceived stigma did not have a significant direct effect on turnover intention (*β* = −0.002, *p* = 0.91).

Concerning autonomous motivation, results indicated that turnover intention exerted a significant negative impact (*β* = −0.34, *p* < 0.001). Perceived stigma also negatively predicted autonomous motivation (*β* = −0.05, *p* = 0.003). Consistent with the reverse‐scoring interpretation, positive response showed a significant negative path to autonomous motivation (*β* = −0.35, *p* < 0.001), suggesting that diminished positive coping is associated with lower autonomous motivation. Neither self‐stigma (*β* = −0.02, *p* = 0.43) nor social concealment (*β* = 0.03, *p* = 0.15) showed a significant direct effect on autonomous motivation.

#### 3.4.2. Indirect Effects

Bootstrap analysis was conducted to examine the indirect effects of stigma dimensions on autonomous motivation via turnover intention (summarized in Table 4). Self‐stigma exerted a significant negative indirect effect on autonomous motivation through turnover intention (*β*_indirect_ = −0.092, 95% CI [−0.120, −0.070]). Given the nonsignificant direct effect, this indicates a full mediation pathway. Similarly, social concealment had a significant negative indirect effect via turnover intention (*β*_indirect_ = −0.028, 95% CI [−0.042, −0.014]), also suggesting full mediation. Positive response showed a significant negative indirect effect (*β*_indirect_ = −0.135, 95% CI [−0.162, −0.112]). Since its direct effect remained significant, turnover intention acted as a partial mediator in this relationship. Finally, the indirect effect of perceived stigma was not significant (*β*_indirect_ = 0.001, 95% CI [−0.012, 0.014]), indicating no mediation effect.

**TABLE 4:** Standardized indirect and total effect analysis.

Paths	Estimate (95% CI, *p* value)	
	Indirect effects	Total effects
Perceived stigma ⟶ turnover intention ⟶ autonomous motivation	0.001 (−0.012∼0.014, 0.930)	−0.050 (−0.085∼−0.011, 0.012)^∗^
Self‐stigma ⟶ turnover intention ⟶ autonomous motivation	−0.092 (−0.120∼−0.070, 0.001)^∗∗^	−0.111 (−0.163∼−0.056, 0.001)^∗∗^
Social concealment ⟶ turnover intention ⟶ autonomous motivation	−0.028 (−0.042∼−0.014, 0.001)^∗∗^	−0.001 (−0.039∼0.034, 0.910)
Positive response ⟶ turnover intention ⟶ autonomous motivation	−0.135 (−0.162∼−0.112, 0.001)^∗∗^	−0.486 (−0.540∼−0.435, 0.001)^∗∗^

Abbreviation: 95% CI, 95% confidence interval.

^∗^ *p* < 0.05.

^∗∗^ *p* < 0.01.

## 4. Discussion

This study examined the associations between multidimensional occupational stigma and autonomous motivation among staff in psychiatric hospitals and tested turnover intention as a potential intervening pathway. The findings indicate that different stigma dimensions relate to autonomous motivation through distinct patterns, offering implications for targeted workforce and nursing management strategies.

Perceived stigma showed a significant negative association with autonomous motivation, consistent with SDT [6, 40]. A plausible interpretation is that perceiving external devaluation may be linked to lower satisfaction of competence and relatedness needs, which may correspond to a shift from more autonomous to more controlled regulation as staff adjust behavior to avoid negative evaluation rather than enact professional values [41–44]. Such experiences may also be associated with weaker professional identity and reduced proactive engagement, consistent with research linking occupational stigma with diminished self‐evaluations and lower engagement [45].

In contrast, self‐stigma and social concealment were not directly associated with autonomous motivation but showed significant indirect associations via turnover intention. This pattern aligns with the ABC framework, in which internalized stigma may be associated with emotional strain and withdrawal cognitions that are reflected in stronger intentions to leave [44, 46–48]. Social concealment, often shaped by perceived external pressures to protect self‐image, may also be linked to reduced access to social support, particularly in collectivist contexts where harmonious relationships are valued [49, 50]. Together, these processes may be reflected in higher turnover intention, which in turn was negatively associated with autonomous motivation.

Positive response represents the behavioral component within the ABC framework. In this study, lower levels of positive response (represented by higher reverse‐coded scores) reflect a heavier burden of stigma. Our findings indicate that this behavioral deficiency is directly associated with lower autonomous motivation, while also indirectly undermining it through the affective pathway of turnover intention. Within the ABC logic, when staff lack proactive behavioral strategies to counter negative cognitive appraisals of stigma, they are more susceptible to emotional strain and withdrawal intentions [44, 46]. Furthermore, the lack of positive behavioral coping directly diminishes the staff’s autonomous drive, as they may feel less capable of maintaining professional values under pressure [47]. This suggests that positive response acts as a dual‐action resource: It not only buffers the transition from cognitive stigma to affective withdrawal but also directly sustains the behavioral persistence of autonomous motivation.

The absence of a significant association between perceived stigma and turnover intention suggests that the relationship between perceived stigma and withdrawal is likely more complex than previously assumed. Perceived external devaluation may not necessarily translate into withdrawal intentions for all staff. Attributional processes, such as attributing negative portrayals to media bias rather than personal shortcomings, may buffer how perceived stigma relates to turnover intention [51, 52]. Additionally, contextual factors such as professional identity strength or organizational support could play moderating roles. These interpretations are preliminary and warrant further examination into potential boundary conditions that shape these relationships.

From a nursing management perspective, the findings suggest the need for differentiated strategies. Efforts to reduce perceived stigma may benefit from organizational advocacy and public engagement to improve societal perceptions of psychiatric care. Interventions addressing self‐stigma and concealment may focus on psychological support, peer mentoring, and strengthening workplace social support. In addition, organizational practices that support positive response (e.g., leadership development, recognition programs, and participatory decision‐making) may be linked to lower turnover intention and stronger autonomous motivation by reinforcing professional identity and value alignment.

### 4.1. Limitations and Future Research

Several limitations should be noted. First, the cross‐sectional design precludes causal inference; thus, longitudinal studies are needed to establish temporal ordering. Second, while the use of validated scales and large multisite sampling strengthened the findings, the reliance on self‐report measures may introduce CMB. Third, although strict quality control was implemented (e.g., attention and logic checks), the exclusion of approximately 22.3% of the initial sample due to invalid responses might limit the generalizability of the findings. Future research should specify and refine data collection procedures to further minimize nonresponse bias.

Lastly, our study population primarily consisted of clinical staff in psychiatric hospitals. While a significant portion of participants also held administrative or management roles (e.g., nurse managers or department heads), we did not further differentiate between purely clinical versus dually assigned administrative duties in our analysis. Given that professional characteristics and administrative stressors may influence the identified mediation pathways, future research should employ more granular role differentiation to assess how specific job functions shape the experience of occupational stigma and its motivational outcomes.

## 5. Conclusion

This study elucidates the complex association patterns between multidimensional stigma, turnover intention, and autonomous motivation among psychiatric hospital staff. Our findings highlight that while perceived stigma is directly linked to diminished autonomous motivation, self‐stigma and social concealment primarily relate to motivational outcomes through the intervening pathway of turnover intention. Notably, the positive response dimension, as a reverse‐scored component of stigma, serves as a significant predictor of both withdrawal cognitions and motivational regulation. These results underscore the necessity for nursing management to move beyond generic support and implement differentiated interventions that address specific stigma dimensions. By fostering organizational advocacy to mitigate external devaluation and providing psychological resources to counter internalized stigma, healthcare institutions can better sustain the autonomous motivation of their workforce and enhance staff retention in mental health settings.

## Funding

This research was supported by the Peking University Health Science Center Medical Education Research Funding Project (Grant no. 2023YB26).

## Ethics Statement

The survey protocol received approval from the Biomedical Ethics Committee of Peking University (IRB00001052‐22175), and all participants provided informed consent.

## Conflicts of Interest

The authors declare no conflicts of interest.

## Data Availability

The data that support the findings of this study are available from the corresponding author upon reasonable request.

## References

[1] Deng Y. , Sun S. , Wu S. et al., Burden and Trends of Mental Disorders in China from 1990 to 2019: Findings From the Global Burden of Disease Study 2019, Social Psychiatry and Psychiatric Epidemiology. (2024) 59, no. 9, 1563–1576, 10.1007/s00127-023-02594-x. 38087123

[2] Huang Y. , Wang Y. , Wang H. et al., Prevalence of Mental Disorders in China: A Cross-Sectional Epidemiological Study, The Lancet Psychiatry. (2019) 6, no. 3, 211–224, 10.1016/S2215-0366(18)30511-X, 2-s2.0-85061671000. 30792114

[3] World Health Organization , Comprehensive Mental Health Action Plan 2013-2030, 2013, World Health Organization, Geneva.

[4] Hu X. , Rohrbaugh R. , Deng Q. , He Q. , Munger K. F. , and Liu Z. , Expanding the Mental Health Workforce in China: Narrowing the Mental Health Service Gap, Psychiatric Services. (2017) 68, no. 10, 987–989, 10.1176/appi.ps.201700002, 2-s2.0-85030635060. 28806895

[5] Thompson K. A. , Modini M. , and Abbott M. J. , Factors Influencing Staff Perceptions of Inpatient Psychiatric Hospitals: A Meta‐Review of the Literature, International Journal of Mental Health Nursing. (2024) 33, no. 6, 1711–1728, 10.1111/inm.13374. 39548669

[6] Deci E. L. and Richard M. R. , Self-Determination Theory: A Macrotheory of Human Motivation, Development, and Health, Canadian Psychology. (2000) 41, no. 4, 227–237.

[7] Ryan R. M. and Deci E. L. , Self-Determination Theory and the Facilitation of Intrinsic Motivation, Social Development, and Well-Being, American Psychologist. (2000) 55, no. 1, 68–78, 10.1037//0003-066x.55.1.68. 11392867

[8] Gagné M. and Deci E. L. , Self‐Determination Theory and Work Motivation, Journal of Organizational Behavior. (2005) 26, no. 4, 331–362, 10.1002/job.322, 2-s2.0-20744446898.

[9] Wulandari A. A. N. and Dara S. R. , Determinants of Employee Performance in Healthcare Organization: The Role of Work Environment, Workload, and Motivation, Journal of Healthcare Leadership. (2023) 15, no. 1, 89–101, 10.58777/hco.v1i1.118.

[10] Trifunović B. , Examining the Impact of Managerial Support on the Performance of Healthcare Organizations—The Mediating Role of Employee Autonomy, Srpski arhiv za celokupno lekarstvo. (2024) 152, no. Suppl 1, 80–586, 10.2298/sarh240424080t.

[11] Veenstra G. L. , Clinical Governance and Healthcare Professionals’ Motivation to Provide Care: A Balancing Act, 2022, University of Groningen, PhD diss 10.33612/diss.211516086.

[12] Deci E. L. and Ryan R. M. , The ‘What ’ and ‘Why’ of Goal Pursuits: Human Needs and the Self-Determination of Behavior, Psychological Inquiry. (2000) 11, no. 4, 227–268, 10.1207/s15327965pli1104_01, 2-s2.0-0034549672.

[13] Veenstra G. L. , Dabekaussen K. F. A. A. , Molleman E. , Heineman E. , and Welker G. A. , Health Care Professionals’ Motivation, Their Behaviors, and the Quality of Hospital Care: A Mixed-Methods Systematic Review, Health Care Management Review. (2022) 47, no. 2, 155–167, 10.1097/hmr.0000000000000284. 32271199 PMC8876425

[14] Thornicroft G. , Rose D. , Kassam A. , and Sartorius N. , Stigma: Ignorance, Prejudice or Discrimination?, British Journal of Psychiatry. (2007) 190, no. 3, 192–193, 10.1192/bjp.bp.106.025791, 2-s2.0-33847706555. 17329736

[15] Link B. G. and Phelan Jo C. , Conceptualizing Stigma, Annual Review of Sociology. (2001) 27, no. 1, 363–385, 10.1146/annurev.soc.27.1.363, 2-s2.0-0035629556.

[16] Jauch M. , Occhipinti S. , and O′Donovan A. , The Stigmatization of Mental Illness by Mental Health Professionals: Scoping Review and Bibliometric Analysis, PLoS One. (2023) 18, no. 1, 10.1371/journal.pone.0280739. PMC9858369 36662889

[17] Hei L. P. , Sun S. W. , Zhao M. M. , and Yuan B. B. , Construction of Occupational Stigma Scale for Staff in Psychiatric Hospitals and Its Relationship with Job Satisfaction, Chinese Journal of Health Policy. (2023) 16, no. 7, 76–82.

[18] Zhang X. , Zhang L. , Xue B. et al., Effort–Reward Imbalance and Well-Being Among Psychiatric Nurses: The Mediating Role of Burnout and Decent Work, BMC Nursing. (2024) 23, no. 1, 10.1186/s12912-024-02301-4. PMC11389592 39256745

[19] Cho H. L. and Huang C. J. , Why Mental Health–Related Stigma Matters for Physician Wellbeing, Burnout, and Patient Care, Journal of General Internal Medicine. (2020) 35, no. 5, 1579–1581, 10.1007/s11606-019-05173-6. 32096078 PMC7210325

[20] Chan S. T. , Khong P. C. B. , and Wen W. , Psychological Responses, Coping and Supporting Needs of Healthcare Professionals as Second Victims, International Nursing Review. (2017) 64, no. 2, 242–262, 10.1111/inr.12317, 2-s2.0-84995377281. 27679402

[21] Riffel T. and Chen S. P. , Stigma in Healthcare? Exploring the Knowledge, Attitudes, and Behavioural Responses of Healthcare Professionals and Students Toward Individuals with Mental Illnesses, Psychiatric Quarterly. (2020) 91, no. 4, 1103–1119, 10.1007/s11126-020-09809-3. 32789718

[22] Tett R. P. and Meyer J. P. , Job Satisfaction, Organizational Commitment, Turnover Intention, and Turnover: Path Analyses Based on Meta‐Analytic Findings, Personnel Psychology. (1993) 46, no. 2, 259–293, 10.1111/j.1744-6570.1993.tb00874.x, 2-s2.0-84993021371.

[23] Poku C. A. , Bayuo J. , Agyare V. A. , Sarkodie N. K. , and Bam V. , Work Engagement, Resilience and Turnover Intentions Among Nurses: a Mediation Analysis, BMC Health Services Research. (2025) 25, no. 1, 10.1186/s12913-025-12242-6. PMC11730472 39806365

[24] Corey G. , Theory and Practice of Counseling and Psychotherapy, 2017, 10th edition, Cengage Learning, Boston.

[25] Ellis A. , Reason and Emotion in Psychotherapy, 1962, Lyle Stuart, New York.

[26] Deci E. L. and Richard M. R. , Self-Determination Theory: A Macrotheory of Human Motivation, Development, and Health, Canadian Psychology/Psychologie canadienne. (2008) 49, no. 3, 182–185, 10.1037/a0012801, 2-s2.0-54049099212.

[27] Liao G. , Liu J. , Li Y. , Ye H. , and Liang J. , Effect of Healthcare Professionals’ Perceived Occupational Stigma on Organizational Citizenship Behavior: A Moral Cleansing Perspective, BMC Medical Ethics. (2025) 26, no. 1, 10.1186/s12910-025-01185-6. PMC11844104 39984970

[28] Stevens J. P. , Applied Multivariate Statistics for the Social Sciences, 2009, 5th edition, Routledge, New York.

[29] Field A. , Discovering Statistics Using IBM SPSS Statistics, 2013, 4th edition, SAGE Publications, London.

[30] Wang F. , Zhang M. , Huang Y. et al., Impact of Psychosocial Factors on Mental Health and Turnover Intention Among Health Workers at Different Occupational Statuses: An Exploratory Cross-Sectional Study in China, European Journal of Investigation in Health Psychology and Education. (2025) 15, no. 5, 10.3390/ejihpe15050073. PMC12110252 40422302

[31] Statistical Information Center of the Ministry of Health , A Study on China’s Doctor-Patient Relationship, 2008, Chinese Journal of Health Informatics and Management, Beijing.

[32] Fornell C. and Larcker D. F. , Evaluating Structural Equation Models with Unobservable Variables and Measurement Error, Journal of Marketing Research. (1981) 18, no. 1, 39–50, 10.2307/3151312.

[33] Podsakoff P. M. , MacKenzie S. B. , Lee J.-Y. , and Podsakoff N. P. , Common Method Biases in Behavioral Research: A Critical Review of the Literature and Recommended Remedies, Journal of Applied Psychology. (2003) 88, no. 5, 879–903, 10.1037/0021-9010.88.5.879, 2-s2.0-0141907688. 14516251

[34] Hu L. and Bentler P. M. , Cutoff Criteria for Fit Indexes in Covariance Structure Analysis: Conventional Criteria Versus New Alternatives, Structural Equation Modeling: A Multidisciplinary Journal. (1999) 6, no. 1, 1–55, 10.1080/10705519909540118, 2-s2.0-67650706330.

[35] Browne M. W. and Cudeck R. , Bollen K. A. and Scott Long J. , Alternative Ways of Assessing Model Fit, Testing Structural Equation Models, 1993, Sage, Newbury Park, CA, 136–162.

[36] Preacher K. J. and Hayes A. F. , Asymptotic and Resampling Strategies for Assessing and Comparing Indirect Effects in Multiple Mediator Models, Behavior Research Methods. (2008) 40, no. 3, 879–891, 10.3758/brm.40.3.879, 2-s2.0-44949168308. 18697684

[37] Thornicroft G. , Sunkel C. , Aliev A. et al., The Lancet Commission on Ending Stigma and Discrimination in Mental Health, The Lancet. (2022) 400, no. 10361, 1438–1480, 10.1016/s0140-6736(22)01470-2. 36223799

[38] Cheung G. W. and Rensvold R. B. , Evaluating Goodness-of-Fit Indexes for Testing Measurement Invariance, Structural Equation Modeling: A Multidisciplinary Journal. (2002) 9, no. 2, 233–255, 10.1207/s15328007sem0902_5, 2-s2.0-79851496916.

[39] Chen F. F. , Sensitivity of Goodness of Fit Indexes to Lack of Measurement Invariance, Structural Equation Modeling: A Multidisciplinary Journal. (2007) 14, no. 3, 464–504, 10.1080/10705510701301834, 2-s2.0-34548107817.

[40] Marpaung Y. M. , Ernawati E. , and Fushen , Unmasking Stigma: A Qualitative Exploration of Nurses in Urban and Rural Indonesia During the COVID-19 Pandemic, The Open Nursing Journal. (2024) 18, no. 1, 10.2174/0118744346313412240603062313.

[41] Richman L. S. and Lattanner M. R. , Self-Regulatory Processes Underlying Structural Stigma and Health, Social Science & Medicine. (2014) 103, 94–100, 10.1016/j.socscimed.2013.12.029, 2-s2.0-84893496681. 24507915

[42] Allan B. A. , Autin K. L. , and Duffy R. D. , Self-Determination and Meaningful Work: Exploring Socioeconomic Constraints, Frontiers in Psychology. (2016) 7, 10.3389/fpsyg.2016.00071. PMC4735399 26869970

[43] Fekonja Z. , Kmetec S. , Novak B. , McCormack B. , and Mlinar Reljić N. , A Qualitative Study of Family Members′ Experiences of Their Loved One Developing Dementia and Their Subsequent Placement in a Nursing Home, Journal of Nursing Management. (2021) 29, no. 5, 1284–1292, 10.1111/jonm.13267. 33484604

[44] Findyartini A. , Greviana N. , Felaza E. , Faruqi M. , Zahratul Afifah T. , and Auliya Firdausy M. , Professional Identity Formation of Medical Students: A Mixed-Methods Study in a Hierarchical and Collectivist Culture, BMC Medical Education. (2022) 22, no. 1, 10.1186/s12909-022-03393-9. PMC9175156 35676696

[45] Zhou Y. , Huang X. , and Ouyang K. , Initiative or Avoidance: Professional Stigma, Self-Evaluation and Task Performance, Foreign Economics and Management. (2020) 42, no. 8, 50–67.

[46] Choi H. , The Impact of Self-Stigma and Self-Determination on Career Outcomes of Transition-Age Individuals With Disabilities: A Structural Equation Modeling Approach, 2024, Michigan State University, PhD dissertation.

[47] Huang B. , Ma L. , and Huang L. , My Work is Meaningless: The Consequences of Perceived Occupational Stigma for Employees in High-Prestige Occupations, Frontiers in Psychology. (2022) 13, 10.3389/fpsyg.2022.715188. PMC9092528 35572310

[48] Van der Goot W. E. , Van Yperen N. W. , Albers C. J. , Jaarsma A. D. C. , and Duvivier R. J. , Effects of (De) Motivating Supervision Styles on Junior Doctors’ Intrinsic Motivation Through Basic Psychological Need Frustration and Satisfaction: An Experimental Vignette Study, Advances in Health Sciences Education. (2024) 30, no. 2, 1–26, 10.1007/s10459-024-10344-0. PMC11965158 38916844

[49] Wang L. et al., Living Alone but Not Feeling Lonely: The Effect of Self-Concealment on Perceived Social Support of Youth Living Alone in China, International Journal of Environmental Research and Public Health. (2025) 22, no. 24. 10.3390/ijerph192113805 PMC9654297 36360684

[50] Kim J. , Examining the Relationships Among Concealment Tendencies, Illness Attitudes, Belief in a Just World, and Cognitive Flexibility, Frontiers in Psychology. (2021) 12, 10.3389/fpsyg.2021.627739. PMC8446418 34539475

[51] Pepermans R. and Peiffer M. , Choosing Jobs in the Public, Non-Profit, and For-Profit Sectors: Personal Career Anchors Moderating the Impact of Sector Image and Reputation, Review of Public Personnel Administration. (2024) 44, no. 2, 295–324, 10.1177/0734371x221130972.

[52] Ji H. , Yan J. , and Guo W. , How and When Does Occupational Stigma Promote Intent to Leave? The Mediation Effect of Family Implicated Stigma and the Moderating Effect of Family Involvement, Acta Psychology Sinica. (2022) 54, no. 2, 182–195.

